# Powerline bioactivity - more than magnetism

**DOI:** 10.1186/2193-1801-2-454

**Published:** 2013-09-11

**Authors:** G Hugh Sidaway

**Affiliations:** 111 Waun Fach Pentwyn, Cardiff, CF23 7BD UK

**Keywords:** Childhood leukaemia, Overhead powerlines, Air ionization, Superoxide, Magnetic fields, Immune dysfunction, Epigenetics, Mexico

## Abstract

**Background:**

Previous work on the possible public health impact of electricity utilization has mostly considered low frequency electromagnetic fields, particularly those associated with high voltage overhead powerlines, but no generally accepted biological mechanism has been proposed. The present study seeks to expand the area of debate to include airborne electroactivity.

**Findings:**

From a literature survey it is concluded that there is statistically significant published evidence consistent with the involvement of airborne electroactive agents in the powerline proximity modulation of some cytokine activity. Attention is drawn to overhead line fault associated corona discharge action as a source of potentially bioactive agents deserving careful study in view of the widespread close residential proximity to overhead power distribution lines in many countries. Particular attention is given to the role of electricity access associated faults as a possible explanation for the high childhood leukaemia rates in certain districts of Mexico City.

**Conclusions:**

Despite more than 30 years research worldwide there is no generally accepted biological mechanism to explain the adverse health impact of overhead powerline residential proximity. Expanding the area of consideration to include airborne electroactivity may provide the basis for a plausible outline model of such a mechanism. More attention should be given to this research area.

**Electronic supplementary material:**

The online version of this article (doi:10.1186/2193-1801-2-454) contains supplementary material, which is available to authorized users.

## Findings

### Powerlines

The public health impact of electricity utilization has been a matter of controversy for over 30 years. At the present time, general awareness of this issue may be enhanced by recent publication of the results of the French *Geocap* study (Sermage-Faure et al. 2013). These authors conclude that their findings support the hypothesis that close residential proximity to a very high voltage overhead line may be associated with an increased incidence of childhood acute leukaemia. Most studies have concerned the electromagnetic field (EMF) aspect- for example, the work of Li et al. (2011) which established a link between childhood asthma and maternal exposure to low-frequency EMFs. These however constitute only one component of the electricity utilization environment. Concentration on electromagnetic fields has diverted attention from the ability of many electrically energized structures to generate air ions by ‘arcing’ even at low voltages :for example the sparking of electromechanical relays in older telephone exchanges operating at less than 100 V (see literature cited in reference 5 (page 307) of Sidaway (2012).

Extending epidemiological considerations to include lower-voltage overhead powerlines, rather than the high magnetic field producing, high voltage lines presently considered would lead to conclusions contrasting sharply with those expressed by Schmeidel and Blettner (Schmiedel and Blettner 2010) that “the public health impact is low”. The “Kaiser Permanente Medical Care Program” (KP) population in California studied in the report by Li et al. (2011) was originally defined by Li et al. (2002) who noted that maximum magnetic field exposure levels were comparable with those found in a nationwide survey (Zaffanella and Kalton 1998) which associated high magnetic field exposure at home with close residential proximity to overhead powerlines (page 6-9, and see page A-56). The purpose of this “1000 Person Survey” was to establish the magnetic field exposure of the general U.S. population by measurements on a population sample, selected by random digit telephone number dialling such that each household selected was considered to represent 23,350 other households not contacted. Although the authors advise that the survey findings should be interpreted with caution, data presented on page 5-51 appear to suggest that around 75% of the U.S. population may live less than 50 m from some form of overhead powerline. The population of the KP study was drawn from an area similar to, and to some extent overlapping with, that covered by the “Northern California Childhood Leukaemia Study” (NCCLS), for example Chang et al. (2009) who found a positive association between childhood leukaemia and maternal immunoglobin status. Web-accesible streetviews (web link Ref 1) suggest a broad similarity with power distribution wiring near apartments in Denver, Colorado shown in a photograph published by Crumpton (2000) to illustrate the type of overhead wiring studied in the pioneering work by Wertheimer and Leeper (1979). Some characteristics of the overhead distribution lines widely used in the U.S. are discussed in literature cited in Sidaway (2012). As evidenced by streetviews (web link Ref 1), similar distribution wiring is widely used in countries such as Australia, China, Japan, Mexico and New Zealand.

### Immune dysfunction

A long-term study of possible high voltage transmission line effects on sheep was initiated in 1990 by *Bonneville Power Administration,* Portland, Oregon. Results of the study were presented in a series of Reports, the last two of which were published by the *Electric Power Research Institute*, which sponsored Phases 4 and 5 of the study. Experimental animals were housed in a pen within a powerline corridor containing 3 x 500 kV and 2 x 230 kV transmission lines. Control animals were some 230 m distant. Phases 2 and 3 of the study found that activity of the cytokine IL-1 was significantly reduced in experimental animals but removing the animals from exposure conditions resulted in a return of cytokine activity to control levels (see Sidaway 2012). The Phase 2 Report (Stormshak 1993) contains (otherwise unavailable) information regarding possible immune dysfunction associated with the development of fungal skin infections in sheep exposed to the powerline environment. Two human infections also occurred in study personnel. These observations are clearly important as Chang et al. (2011) noted that childhood acute lymphoblastic leukaemia may be associated with a dysregulated immunefunction. Il-1 has an important role in regulating defence mechanisms against pathogens including fungi (Ben-Sasson et al. 2009; Sims and Smith 2010). During Phases 4 and 5, a second experimental pen was added in which animals were shielded from the powerline electric field by grounded metal screens (see Sidaway 2012). A summary of parts of Reports 4 and 5 was published by Hefeneider et al, (Hefeneider et al. 2001) who noted a significant correlation between wind direction and IL-1 activity. This would be consistent with the action of powerline-associated airborne bioactive agents.

Respiratory airway epithelial cells are positioned to monitor the ambient atmospheric environment and detect and respond to airborne potential pathogens via pathogen structure pattern recognition receptors (Kato and Schleimer 2007). It is possible that epithelial cell function could be modulated by airborne electric charge carrying agents, leading to changes in cytokine activity. It is one purpose of the present communication to suggest that such agents could act as environmental cues in the context of the “developmental origin of health and disease” model of human developmental plasticity (Hochberg et al 2011) and could thus be relevant to the suggested association between childhood asthma and maternal magnetic field exposure (Li et al. 2011). The same (or related) electro-environmental cues could drive the association between electricity utilization and some childhood cancers - note Chang et al. (2011) from the NCCLS area. As pointed out by Sidaway (2012), unlike EMFs, air ions occur naturally, and cover such a broad structural spectrum as to make it difficult to specify, with certainty, the precise nature and origin of putative causative agents. By clouding the issue of adverse health impact hazard attribution, the model presented here should reduce the risk of a confrontational approach to public health concerns. An apparent association between health impacts and air ionization, whether derived from high voltage corona activity or more general fault-associated electrical arcing, could justify the exploration and perhaps introduction of a range of precautionary mitigation/prevention measures. The costs of any such measures need to be offset against present and predicted healthcare economic burdens. In purely economic terms, a possible reduction in adverse public health impacts should justify consideration of financial resource redistribution via subsidies.

### Environmental epigenetics

Goldstein et al. (1992) found that the biological activity of negative air ions involves the superoxide anion-radical, and suggest a “prominent ecological and evolutionary” role for exogenous superoxide. In this context note that Esteller (2007) proposed that hypermethylation of tumour suppressor genes may be related to the level of exposure to external carcinogen agents. Biologically important reactions of the superoxide radical are discussed by Maynard et al. (2009) who note that DNA bases, particularly guanine, are sensitive to damage by reactive oxygen species. Valinluck et al. (2004) consider the role of guanine oxidation in epigenetic signal propagation, while Turrens (2003) discusses the endogenous production of superoxide anion and notes the importance of this radical as a precursor of other reactive oxygen species, particularly hydroxyl radical. The potential consequences of electric charge-carrying agent inhalation need to be carefully considered in the context of hydrogen peroxide formation and diffusion (Goldstein et al. 1992; Maynard et al. 2009). Note also the reported association (Yang et al. 2008) between the residential powerline environment and polymorphisms of the DNA repair gene *XRCC1*.

### Mexico

Perez-Saldivar et al (2011) note that the frequency of childhood acute Lymphoblastic leukaemia in Mexico City is among the highest in the world. These authors also note that the standardized average annual incidence rate for childhood leukaemia was highest in the “relatively affluent” Mexico City borough of Cuauhtemoc. It should be noted that, according to To web link Ref 2, “Street vendors have proliferated in Cuauhtemoc”. This is important as illegal electricity access by street vendors is widespread in Mexico City (see literature cited in Sidaway (2012) and could thus increase the frequency of potentially faulty overhead powerline connections in Cuauhtemoc and perhaps some other areas. See also Additional files 1 and 2, and consider the findings of Yang et al. (2008) regarding a possible association between the residential powerline environment and polymorphisms of the DNA repair gene *XRCC1*.

## Electronic supplementary material

Additional file 1: Bioactivity of Electricity Utilization – Broadening the Debate Part 1 – Epidemiology. (DOC 58 KB)

Additional file 2: Bioactivity of Electricity Utilization - Broadening the Debate Part 2 - Biology. (DOC 57 KB)

## Abbreviations

EMF: Electromagnetic field
KP: Kaiser Permanente Medical Care Program
NCCLS: Northern California childhood leukemia study.
