# Supplementary material for: Powerline bioactivity - more than magnetism
Source: Springerplus. 2013 Sep 11;2(1):454. doi: 10.1186/2193-1801-2-454 (PMC3777017; doi:10.1186/2193-1801-2-454)
Supplement: Supplementary file 1 — Additional file 1: Bioactivity of Electricity Utilization – Broadening the Debate Part 1 – Epidemiology. (DOC 58 KB) [file 40064_2013_514_MOESM1_ESM.doc]

| **Bioactivity of Electricity Utilization – Broadening the Debate**  **Part 1 – Epidemiology** |
| --- |
| G Hugh Sidaway |
| 111 Waun Fach, Pentwyn, Cardiff CF23 7BD [derlwyn8@ntlworld.com](mailto:derlwyn8@ntlworld.com)  Much previous work on the public health impact of electricity utilization has considered possible influences of low-frequency electromagnetic fields (EMFs). These however constitute only one component of the global electricity utilization network. Regarding the network solely as a producer of non-ionizing electromagnetic radiation has diverted attention from the ability of many electrically energized structures to generate air ions by transient or extended corona discharge action even at low voltages. The extensive, but now little known, early work of the UK Electroculture Committee was based on air ionization effects on plant growth : magnetic field influences were not even considered. There is now statistically significant published evidence for the involvement of airborne electroactivity in at least one animal response to a.c. transmission line proximity, although the agent(s) concerned have not been identified. Occasional, but sometimes strong, corona activity due to various faults on overhead lines at voltages at least as low as 12.47kV is illustrated on a commercial corona detection camera website which reports such activity to be particularly noticeable in high humidity conditions. 12.47kV is a widely used primary voltage in North-American type distribution systems and easily web-accessible streetviews illustrate many residential areas supplied in this way. Over 50 years ago powerline corona was reported to be detectable near transmission towers and poles and may be particularly associated with insulator breakdown at high atmospheric humidity. Powerline connection density may be an important factor linking population density with putative health impacts. A recent study of childhood leukaemia in relation to power frequency magnetic fields in Japan specifically mentions the presence of “distributing transmission lines to the residences”. In the context of an association between atmospheric humidity and powerline corona, the use of Single Wire Earth Return (SWER) systems for rural electrification in developing countries should be examined more closely in view of their application in Australia and particularly New Zealand which have higher childhood leukaemia rates than the UK.  Mexico City is reported to have one of the highest incidence rates (IR) of childhood leukaemia in the world – a rate which appears to have more than doubled in the last 30 years. Mexican national household access to electricity has also increased greatly over this period and there is therefore a trend comparable with that reported for the increased IR of childhood leukaemia accompanying rural electrification in the UK and USA. The high Mexico City IR however contrasts with geographical incidence in the UK where the rate is presently highest in rural areas with relatively low population density. These UK areas are more likely to be associated with residential overhead wire electricity distribution and supply : most UK urban distribution is via underground cables. As in many developing countries, overhead distribution characterizes much of Mexico and is particularly important in Mexico City where illegal electricity access, for example by street vendors, is widespread and may increase the frequency of potentially faulty line connections. |

Email your abstract **as a Word document** to [conference@childrenwithcancer.org.uk](mailto:conference@childrenwithcancer.org.uk) by 5pm on Thursday 12^th^ January 2012.
