# Supplementary material for: Powerline bioactivity - more than magnetism
Source: Springerplus. 2013 Sep 11;2(1):454. doi: 10.1186/2193-1801-2-454 (PMC3777017; doi:10.1186/2193-1801-2-454)
Supplement: Supplementary file 2 — Additional file 2: Bioactivity of Electricity Utilization - Broadening the Debate Part 2 - Biology. (DOC 57 KB) [file 40064_2013_514_MOESM2_ESM.doc]

| **Bioactivity of Electricity Utilization - Broadening the Debate**  **Part 2 - Biology** |
| --- |
| G Hugh Sidaway |
| 111 Waun Fach, Pentwyn, Cardiff CF23 7BD [derlwyn8@ntlworld.com](mailto:derlwyn8@ntlworld.com)  UK electricity industry measurements reported in 2002 recorded several thousand air ions per cubic cm at a distance of 50m from a High Voltage overhead line. These levels are within the range previously found to be bioactive in work at the University of California. The bioactivity of such air ions remains a matter of controversy – recent work at Bristol has focused on possible indirect action via ion attachment to pre-existing environmental pollutants. Direct ion influences should also be considered however as corona action is known to produce the superoxide anion, providing a link with present interest in epigenetic gene expression regulation. Studies of the possible role of corona-derived superoxide or other reactive species in the inactivation of tumour suppressor genes could provide the basis for one plausible mechanism to interpret the association of powerlines with childhood cancer.  Accepting that air ions may be bioactive introduces a new dimension to recent laboratory-based EMF experiments, many of which have depended on the use of Helmholtz coil equipment. Earlier workers in this field pointed out that apparent biological responses to magnetic fields may actually reflect magnetic influences on some other agent(s), which could include airborne electroactivity. The results of some recent cell culture experiments are consistent with the accelerated movement of slow moving bioactive agents (“ions”) from the atmosphere above the cell culture into the culture medium following MGF activation. Once established however, The MGF may deflect air ion producing agents. Intermittent EMF exposure could thus repeatedly enhance bioactive agent movement from replenished atmospheric reservoirs.  Recently, studies of apparent magnetic responses in the plant  *Arabidopsis thaliana* were submitted to the “gold standard” of independent replication which failed to confirm the original findings. There were however important differences in exposure conditions between the two sets of experiments – the replications at Oxford were performed in controlled environment chambers, unlike the original studies at Frankfurt and Paris where MGF-producing Helmholtz coils stood on laboratory benches in air-conditioned rooms. Air ionization levels were not recorded in any of these reports, but could have differed, particularly in respect of polarity. Overall, this work could be seen as providing support for a possible airborne electroactivity interpretation. Although not directly relevant to childhood cancer, experiments demonstrating the apparent protective influence of radiofrequency EMF exposure against Alzheimers disease may also imply a role for airborne electroactivity in aetiology as only the experimental animals were confined within a Faraday cage, which could influence air ion levels. In this context it should be noted that long-term studies, as part of the US National Toxicology Program, found several cases of EMF environment protection against cancer in experimental animals.  In summary : this may be an area where we need to apply Occam`s Razor. Air ionization **is** biologically active – why not try building on that instead of developing abstruse magnetic hypotheses ? |

Email your abstract **as a Word document** to [conference@childrenwithcancer.org.uk](mailto:conference@childrenwithcancer.org.uk) by 5pm on Thursday 12^th^ January 2012.

**Any abstracts that do not conform to the required format will not be accepted.**

Delete these instructions to allow more space.
